# Heterotopic cervical pregnancy: Case report and literature review

**DOI:** 10.1111/jog.15193

**Published:** 2022-02-21

**Authors:** Yazhen Fan, Aijun Du, Yinfeng Zhang, Nan Xiao, Yunshan Zhang, Junfang Ma, Wenjia Meng, Haining Luo

**Affiliations:** ^1^ Center for Reproductive Medicine Tianjin Central Hospital of Gynecology Obstetrics Tianjin China

**Keywords:** case reports, cervical pregnancy, ectopic pregnancy, heterotopic pregnancy, infertility

## Abstract

Cervical pregnancy (CP) is a rare form of ectopic pregnancy (EP) in which the embryo implants and grows inside the endocervical canal. Heterotopic cervical pregnancy is an even rare form of EP, in which at least two embryos are simultaneously implanted in different sites and only one in the uterine cavity. Although many treatment approaches are available, the ideal management remains unclear. Here, we describe two cases of CP caused by assisted reproductive technologies (ART). One case underwent fertilization with intracytoplasmic sperm injection (ICSI) for male factor infertility, and the other was frozen–thawed embryo transfer (FET) following conventional in vitro fertilization (IVF). Both cases were successfully treated with ultrasound‐guided cervical pregnancy aspiration, and intrauterine pregnancies were effectively protected. To the best of our knowledge, these two were rare case reports use aspiration without additional methods and intrauterine pregnancy achieved live birth.

## Introduction

Cervical pregnancies (CP) are characterized by implantation in the uterine endocervix canal. The incidence of CP has been reported to be about 1:1000 to 1:18 000 pregnancies, accounting for less than 1% of ectopic pregnancies.^1^ Cervical implantation is associated with previous cesarean section delivery, dilation and curettage procedures, and assisted reproductive technologies (ART). The significantly increased risk of bleeding in women with CP, which might be due to the richness of cervical blood vessels and fibrous tissue, might eventually result in hysterectomy or even death of patients.^2^ Therefore, early diagnosis and treatment and reducing the risk of bleeding are essential for women, especially those who wish to preserve their fertility.

Despite improvements in ultrasound technology, the early diagnosis remains challenging owing to its asymptomatic nature in the initial stage. Moreover, due to the rarity of heterotopic CP and the variety in clinical presentation, no guidelines exist for its management. Most of the related literature are case reports describing several methods for the treatment of heterotopic CP, including aspiration, cervical curettage, forceps extraction, cervical cerclage, Foley catheter placement, and local injection of potassium chloride (KCl) or methotrexate.

Here, we present a case study of the management of heterotopic cervical pregnancy. We also present the results of a literature review comparing our novel method with previously described techniques. Written informed consent for the publication of this case report was obtained from the patients.

## Case Report

### Case 1

**FIGURE 1:**
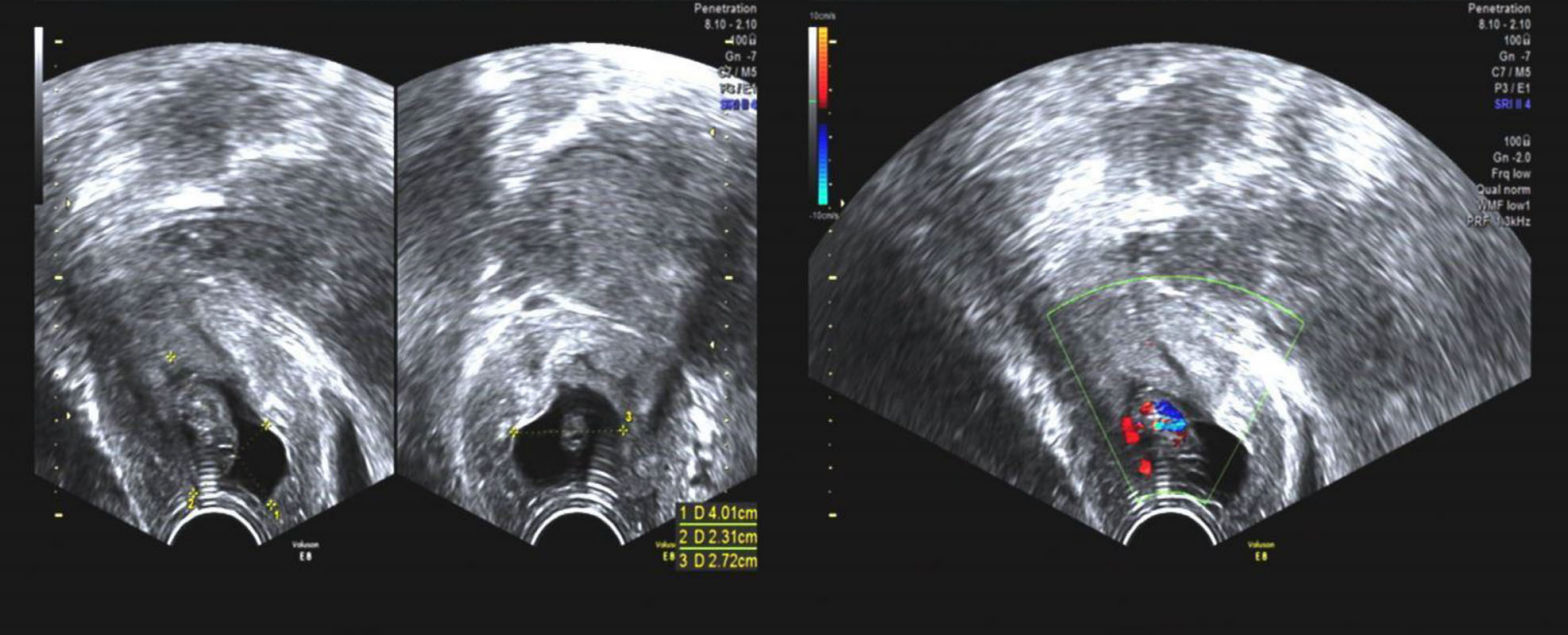
Case 1 ultrasound imaging of cervical pregnancy at the time of diagnosis

A 29‐year‐old nulliparous woman was referred to our center 2 weeks after embryo transfer (ET) with two cleavage‐stage embryos by intracytoplasmic sperm injection (ICSI) for the asthenospermia reason of her husband. The serum beta HCG (β‐HCG) was 819 mIU/mL, with no vaginal bleeding. Fourteen days later, an ultrasound identified a single 14 mm intrauterine gestational sac with a heartbeat. For her β‐HCG 14 days after ET was higher than the average of single pregnancy and lower than twin pregnancy, this woman was counseled to go to the emergency once vaginal bleeding or abdominal pain presented, as two embryos transferred, but only one gestational sac was seen. Transvaginal ultrasound examination 11 days later revealed “threatened abortion” when she was admitted to an emergency with a symptom of vaginal bleeding for 1 day. She rejected further gynecological examination, discharged with treatment for preventing miscarriage only. Three days later (42 days after ET), she was readmitted to an emergency for vaginal bleeding and dizziness. Besides the identified intrauterine gestational sac, another gestational sac in the cervical canal was found by ultrasound, with the size about 40 × 23 × 27 mm. The gestational sac demonstrated a blood flow with a normal fetal heart rate, detected by transvaginal color Doppler examination (Figure 1). Below the gestational sac, there was an attenuation area and loose mass. Gynecological examination showed that gestational tissue was bulging out of the cervical external orifice with bleeding. At the same time, a blood test showed that the hemoglobin was 61 g/L.

Combining the ultrasound, physical examination, and blood test, we considered the diagnosis of heterotopic CP. The patient and her husband had a solid wish to conserve the intrauterine pregnancy as the patient was nulliparous. After being counseled on the risks of different treatment options, they chose conservative treatment. The CP aspiration was performed under the guidance of ultrasound, followed by absorbable hemostatic gauze which was soaked with tranexamic acid used to oppress the cervix to stop bleeding. Intraoperative blood loss was about 40 mL. There was no vaginal bleeding 40 min later. As the hemoglobin was very low, she was given an 800 mL of blood transfusion. Twenty‐four hours later, a repeated blood test showed hemoglobin was 86 g/L. Two days later, another ultrasound was performed, which showed one gestational sac in the uterine with heartbeat, the CRL was 30.6 mm, and she was discharged. The IUP (intrauterine pregnancy) continued without complications. No vaginal bleeding was observed during pregnancy follow‐up. Owing to the fear of CP history will make it difficult for her to deliver through vagina, the patient chose cesarean section without obstetrics indication even though we advised natural delivery. Therefore, she underwent an uncomplicated cesarean section delivery at 39 weeks of a healthy female infant weighing 2750 g, with an Apgar score of 10 in 1 min. The patient did not suffer any postpartum complications.

### Case 2

A 27‐year‐old gravida 1 para 1 woman was admitted to our center 14 days after ET. The woman had undergone a right salpingectomy 4 years ago. Two years ago, she received ART treatment and delivered a female infant. We took hormone replacement treatment (HRT) protocol to prepare endometrium in this FET cycle. Two cleavage stage embryos (8I;8I) were transferred. Serum β‐HCG was 910 mIU/mL 14 days after ET, without vaginal bleeding. Two weeks later (28 days after ET), transvaginal ultrasound showed only one gestational sac in the uterine with a heartbeat. With the experience of previous patient and her β‐HCG was also on the higher side, we advised her to come back in case of any symptoms of vaginal bleeding and abdominal pain. Eight days later (36 days after ET), she was referred with the symptoms of mild vaginal bleeding.

**FIGURE 2:**
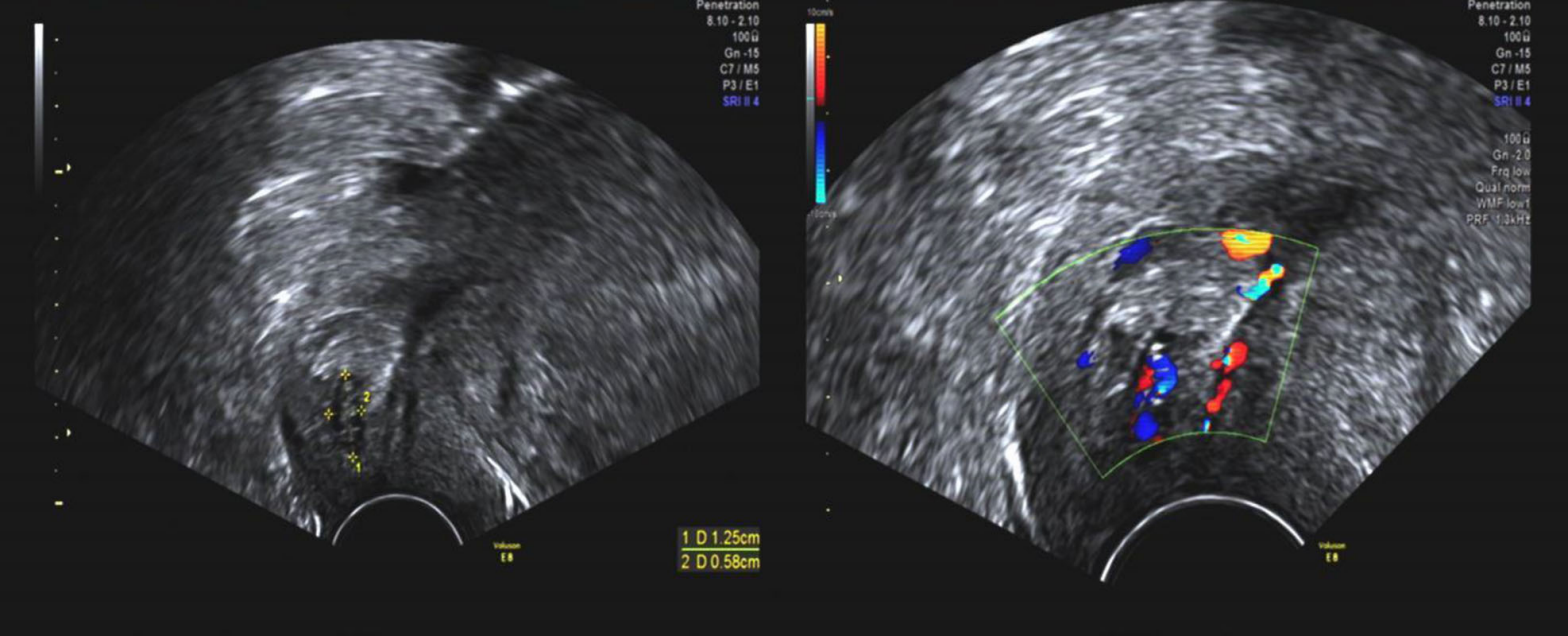
Case 2 ultrasound imaging of cervical pregnancy at the time of diagnosis

Ultrasound examination by an experienced ultrasound expert was conducted, besides the intrauterine gestational sac, there was a 12.5 × 5.8 mm abnormal echo in the cervical canal. Both of them had heartbeat and blood flow signals (Figure 2). Blood test showed that the hemoglobin was 125 g/L, the suspected diagnosis of heterotopic cervical pregnancy was confirmed (Figure 2). After counseling treatment options and risks, they preferred ultrasound‐guided aspiration to preserve the intrauterine pregnancy. The procedure lasted for 20 min, and blood loss was about 20 mL, then we oppressed the cervix with tranexamic acid‐soaked absorbable gauze for 20 min. Hemoglobin after aspiration was 121 g/L. The patient was discharged 24 h later. Ten days later, ultrasound showed one gestational sac in the uterine and CRL was 22.3 mm, with a heartbeat, and there was no abnormal echo in cervical canal. No vaginal bleeding was observed during pregnancy follow‐up and no other complications were found during gestation period inspection. She had a vaginal delivery of a male infant at 27 weeks because of premature rupture of membrane (PROM). The infant weight was 1240 g, and an Apgar score was 7 in 1 min. After 100 days of treatment in the neonatology department, the newborn was discharged with a bodyweight of 3600 g.

## Discussion

Here, we describe the diagnosis and treatment of two cases with heterotopic CP after ART. They were all treated with ultrasound‐guided aspiration. For the first case, it took a long time to confirm the diagnosis of heterotopic CP. The patient had relatively severe bleeding. Fortunately, the outcomes for her and the baby were satisfactory. With the experience of the first patient, we promptly diagnosed heterotopic CP of the second case, which results in minor blood loss. During the treatment, we also have conducted a literature review of CP diagnosis and treatment.

The prevalence rate of CP is rarely low. It has been reported that 50%–90% of the patients have a history of uterine curettage.^3^ Other potential predisposing risk factors have also been identified, including endometritis, altered intrauterine environment, intrauterine device (IUD), uterine deformities, uterine fibroids, congenital fetal abnormalities, and ART.^4^ The increased incidence of ectopic pregnancies following IVF remains inadequately explained. Multiple embryos were transferred during IVF, which might increase the possibility of multiple embryo implantation. Other reasons may be the detrimental effect of supra‐physiological hormone level during ovarian stimulation on endometrial receptivity.^5^ Chen et al. believed that infertility itself and the cervical damage and embryo reflux caused by ET would be risk factors.^6^ Li et al. believed that transferring a day 5 blastocyst in a frozen embryo could reduce the incidence of complex pregnancy.^7^ The possible reason was that in natural pregnancy, embryo implantation occurs on days 5–6 after fertilization, endometrium, and embryo were in a more natural state in the frozen–thawed cycle. Previous studies also reported that different fertilization ways could influence cervical pregnancy rates. ICSI was considered associated with lower CP rates than conventional IVF, which may be caused by an unknown denudation effect of ICSI before fertilization.^8^ Two cases of heterotopic CP were reported in this study, one case was ICSI‐ET, and the other was FET pregnancy. During the transplantation process, the uterine cavity may be stimulated, resulting in abnormal uterine contraction, and the embryo moved down to the cervical implantation.

At present, the commonly used diagnostic methods are Doppler ultrasound, magnetic resonance imaging (MRI), and so on. Ultrasound was the most commonly used auxiliary examination of cervical pregnancy. In 1987, Hofmann et al. described the CP diagnosis criteria by ultrasound as follow^1^: an echo‐free uterine cavity or absence of the fetal parts inside the uterine cavity^2^; hour‐glass uterine shape^3^; ballooned cervical canal^4^; gestational sac in the endocervix, with or without fetal structures^5^; placental tissue in the cervical canal; and^6^ closed internal orifice.^9^ However, CP needs to be distinguished from intrauterine pregnancy that slips to the cervix. Jurkovic et al. believed that intrauterine pregnancy with abortion to the cervical level could show a “sliding sign” by transvaginal ultrasound. That was, the gestational sac could slide to the top of the cervical canal when gently pressing the vaginal ultrasound probe, while cervical pregnancy does not have this feature.^10^ The two cases we reported in this paper, we know that they had a history of ART, ultrasound was performed more carefully and patiently, they were both diagnosed in this way successfully. MRI can be used as a supplementary examination to make a definite diagnosis when ultrasound was not enough. Jung et al. believed that the MRI of CP includes gestational sac occupying the cervix, normal endometrium, hourglass‐shape uterus, and enlarged cervical canal.^11^ MRI could show the location, size, range, signal characteristics, and infiltration degree of cervical pregnancy better, which was of high value for diagnosing complex cases. The cases reported in this paper, combined with the patient's history and ultrasound results, were well diagnosed, so MRI was not adopted.

Because of the rarity of CP, there is no conclusion on the best treatment to date. The treatment choice should be made based on gestational age, initial serum β‐HCG level, presenting fetal heartbeats, vaginal bleeding, and the patient's willingness to preserve fertility. At present, the methods used for the treatment of CP include drug therapy, uterine artery embolization (UAE), ultrasound‐guided cervical pregnancy clearance, and so on. Conservative treatment of drugs could lead to the natural excretion of pregnancy by its cytotoxic effect. Research by Stabile et al. suggested that drug treatment was not related to the size of the gestational sac but the value of serum β‐HCG. The higher the value of serum β‐HCG, the higher the risk of drug treatment failure and subsequent massive hemorrhage.^12^ In addition, Kao et al. believed that gestational time > 9 weeks, serum β‐HCG ≥5000 mIU/mL, and CRL >10 mm would contribute to the drug failure.^13^ The frequently used drug for CP was methotrexate, followed by mifepristone and misoprostol. Systemic treatment of methotrexate might cause serious complications, including nausea, stomach discomfort, diarrhea, hepatotoxicity, myelosuppression, and so on.^14^ Topical methotrexate was seldom in clinical practice. Verma et al. reported two patients with complicated intrauterine CP having a full‐term delivery via topical injection of methotrexate in combined with potassium chloride to kill embryos.^15^ However, its long‐term effect on gestation in intrauterine pregnancy was not clear. Moreover, due to the limited efficacy of drug treatment, Tanos et al. believed that about one‐third of patients who have received conservative drug treatment still need further surgery to treat CP thoroughly.^16^ The two cases reported in this study were intrauterine combined with CP, considering that the topical application of the drug may also have a small amount absorbed into the blood circulation, which may have an adverse effect on intrauterine pregnancy, so we do not recommend this method. Another method UAE could be used for emergency hemostasis of vaginal bleeding to avoid hysterectomy and protect fertility by widely blocking uterine blood vessels. However, extensive UAE could also have side effects, such as endometrial atrophy, secondary amenorrhea, uterine necrosis, and in severe cases, impaired ovarian function, and premature ovarian failure.^17^ The two patients with infertility reported in this study all have a strong will of fertility preservation. UAE might damage the function of the ovary and endometrium. Therefore, patients with infertility are not recommended to use this method.

In this report, we recommend the method of aspiration under the guidance of ultrasound. Cervical pregnancy aspiration under ultrasound can be performed quickly and effectively without damaging the intrauterine gestational sac. We conducted a PubMed‐searching for the terms of “cervical pregnancy,” “heterotopic pregnancy,” and “aspiration,” finding 14 case reports of CP treated with aspiration. These reports vary from the year 1980 to 2019 (Table 1, ^1^, ^6^, ^18^, ^19^, ^20^, ^21^, ^22^, ^23^, ^24^, ^25^, ^26^, ^27^, ^28^, ^29^). The median diagnosis age of these maternal cases was 32 (range from 25 to 41) years. The time to the first diagnosis was from 25 days after ET to 9 weeks of pregnancy. Among these patients, 57.1% (*n* = 8) resulted from IVF/ICSI, 7.1% (*n* = 1) became pregnant after ovulation induction with clomiphene citrate. Half (*n* = 7) of them was asymptomatic when first detected and 71.4% (*n* = 10) are heterotopic CP. All of them were trying their best to maintain intrauterine pregnancy. Eight of them succeeded in having term deliveries, and one was still pregnant at the time of reporting; another was abortion 1 day after aspiration. By pooled analysis of all the case reports, only 35.7% (*n* = 5) of patients underwent aspiration alone. The others were treated with other additional options, including cerclage (*n* = 4), curettage (*n* = 3), Foley catheter insertion (*n* = 2), methotrexate (*n* = 2), KCL (*n* = 1), hypertonic solution sodium chloride (*n* = 1), and glucose (*n* = 1). Some cases with two or more of these mentioned methods. Of the patients underwent aspiration alone, three had live birth,^19^, ^23^, ^25^ the other two cases were abortion after the procedure.^18^, ^29^ In this study, ultrasound‐guided aspiration was conducted by us, followed by oppressing the cervix with tranexamic acid‐soaked absorbable hemostatic gauze to stop bleeding. There was only minimal postoperative bleeding without any other complications. We highlight the importance of oppress after aspiration, which is of the best economic efficiency for patients. For the first patient, oppression after aspiration lasted for 40 min, during the whole process, we did not use any other interventions. The patient endured the process well and bleeding was effectively stopped. During the follow‐up, the two patients did not have other symptoms of vaginal bleeding, and both had a live birth.

**TABLE 1:** Literature review of heterotopic cervical pregnancy treated with aspiration

Article	Age	Pregnancy way	Medical history	Symptom	Embryos implanted	Embryo morphology	Time to first detect	Treatment	Pregnancy outcome
Drezett et al.^18^	36	IVF	G0P0. hyperprolactinemia	Vaginal bleeding	One in the cervix	Cleavage embryo	36 days after ET	Aspiration under laparoscopy	Intentional abortion
Chen et al.^6^	35	ICSI	Bilateral salpingectomy polypectomy	Mild vaginal bleeding	One intrauterine and one in the cervix	Cleavage embryo	25 days after ET	Aspiration, local injection of KCl, cerclage (10 weeks)	Live birth through cesarean section at 38 weeks
Shah et al.^19^	34	IVF/ICSI	G4P2Myomectomy cesarean section curettage	Asymptomatic/painless vaginal bleeding after aspiration	One intrauterine and one in the cervix	Cleavage Embryo	34 days after ET	Aspiration/internal iliac artery balloon catheters placement before delivery	Live birth through cesarean section at 37 weeks
Cepni I et al.^20^	27	Spontaneous	unremarkable	vaginal bleeding, lower abdominal pain, and nausea	one in the cervix	—	7 weeks	Aspiration and systematic methotrexate	Intentional abortion
Prorocic and Vasiljevic^21^	31	IVF	Bilateral sal‐pingectomy	Vaginal bleeding	Two in the uterine and one in the external cervical ostium	Not mentioned	6 weeks	Aspiration and local injection of hypertonic solution of sodium chloride	Ongoing pregnancy
Tsakos et al. ^1^	41	IVF	G2P0 History of ectopic cer‐vical pregnancy	Asymptomatic	One intrauterine and one in the cervix	Blastocyst‐stage embryo	7 weeks+ 5days	Aspiration, Foley catheter insertion, cerclage	Live birth through cesarean section at 38 weeks
Kim et al.^22^	30	Spontaneous	Not mentioned	Asymptomatic/bleeding during aspiration	one intrauterine and one in the cervix	—	8 weeks	Aspiration and Foley catheter insertion	Live birth through cesarean section at 37 weeks
Ujvari et al.^23^	27	IVF	G0P0 bilateral occlusion of uterine tubes	Asymptomatic	Two intrauterine and one in the cervix	Cleavage embryo	4 weeks after ET	Aspiration	Two live birth through cesarean section at 29 weeks
Giorgio et al.^24^	30	Spontaneous	Not mentioned	Painless vaginal bleeding	One in the cervix	—	9 weeks pregnancy	Suction aspiration curettage and suture	Intentional abortion
Cho et al.^25^	35	IVF	Not mention	Asymptomatic	One intrauterine and one in the cervix	Not mention	7 weeks	Aspiration	Delivery of a healthy infant at 35 weeks
Centini et al.^26^	36	Spontaneous	cesarean section	diffuse pains in the lower pelvis	One in the cervix	—	6 weeks+ 2 days	Aspiration, curettage, and methotrexate	Intentional abortion
Faschingbauer et al.^27^	25	clomiphene citrate/spontaneous	Unremarkable	vaginal bleeding	One intrauterine and one in the cervix	—	9 weeks	Aspiration and cerclage	Live birth through vaginal delivery at 39 weeks+3 days
Suzuki et al.^28^	35	IVF	Unremarkable	Asymptomatic	Two intrauterine and one in the cervix	Not mention	24 days after ET	Aspiration and local injection of 33% glucose solution	Two live birth through cesarean section at 34 weeks
Porpora et al.^29^	29	Spontaneous	Left sal‐ pingectomy	asymptomatic	One intrauterine and one in the cervix	—	6 weeks	Aspiration	Abortion (1 day after aspiration)

Abbreviations: ET, embryo transfer; ICSI, intracytoplasmic sperm injection; IVF, in vitro fertilization.

In summary, the prognosis of patients with CP depends on early diagnosis. Considering with patients' medical history, clinical manifestations, and related examinations, a diagnosis can be made. Ultrasound is firstly recommended for diagnosis at present. MRI can identify patients who are difficult to be diagnosed by ultrasound. Especially for patients with two embryos transferred by IVF/ICSI‐ET or FET, attention should be paid to the appearance of the gestational sac outside the uterus in the process of ultrasound. Cervical pregnancy aspiration under the guidance of ultrasound for early detected heterotopic CP is recommended to protect the intrauterine pregnancy effectively.

## Data Availability

The data that support the findings of this study are available from the corresponding author upon reasonable request.
